# Simultaneous Percutaneous Interventional Treatment of Atrial Septal Defects and Pulmonary Valve Stenosis in Children Under the Guidance of Transoesophageal Echocardiography Alone: Preliminary Experiences

**DOI:** 10.3389/fcvm.2022.771281

**Published:** 2022-01-24

**Authors:** Xuning Lu, Ping Wen, Yuhang Liu, Quanwei Zhu, Ning Wang

**Affiliations:** Heart Center, Dalian Municipal Women and Children's Medical Center (Group), Dalian, China

**Keywords:** pulmonary valve stenosis, atrial septal defect, interventional treatment, transoesophageal echocardiography, children

## Abstract

**Objective:**

To investigate the efficacy and safety of simultaneous percutaneous interventional treatment of atrial septal defects (ASDs) and pulmonary valve stenosis (PS) in children under the guidance of transoesophageal echocardiography (TEE) alone.

**Methods:**

Eleven children with ASD combined with PS who were treated at our hospital between March 2015 and March 2019 were recruited, including 4 males and 7 females. Preoperative transthoracic echocardiography showed that all patients had type II ASDs of the foramen ovale subtype, with a maximum diameter of 12.9 ± 2.7 mm (9.0–18.0 mm). The guiding principle of septal occluder selection is that the diameter of the occluder should be 2–4 mm larger than the maximum diameter of the ASD. The pressure gradient across the pulmonary valve in patients with PS was 54.7 ± 5.8 mmHg (47.0–64.0 mmHg), and a balloon with a diameter 1.2–1.4 times the diameter of the pulmonary valve annulus was used for dilatation. Effective dilatation was repeated 2–3 times. All children underwent ASD occlusion and PS balloon dilatation through the femoral vein under TEE guidance without radiation or contrast agents. The patients underwent PS balloon dilatation first, followed by ASD occlusion. The treatment effect was evaluated by TEE immediately after the procedure, and the patients were followed up regularly.

**Results:**

All patients underwent successful simultaneous ASD occlusion and PS balloon dilatation through the femoral vein under the guidance of TEE alone. The pressure gradient across the pulmonary valve immediately after the procedure was 21.3 ± 1.8 mmHg (19.0–25.0 mmHg) (*P* < 0.01). No shunt was detected at the atrial septum level. The patients were followed for 3.0 ± 1.4 years (1.0–5.0 years) after the procedure. The atrial septal occluders were in the normal position in all of the patients, and there was no arrhythmia, hemolysis, or residual shunting. The pressure gradient across the pulmonary valve at 1 month after the procedure was 18.5 ± 3.3 mmHg (*P* < 0.01).

**Conclusion:**

Simultaneous percutaneous interventional treatment of ASD and PS in children under the guidance of TEE alone is not only safe and effective but also prevents trauma caused by extracorporeal circulation and surgical incision and damage caused by X-ray and contrast agents. The surgical sequence included first performing PS balloon dilatation, followed by ASD occlusion.

## Introduction

Congenital heart disease is commonly complicated with malformations. Currently, the main treatments for patients with atrial septal defects (ASDs) combined with pulmonary valve stenosis (PS) are conventional surgery and vascular intervention (1). Conventional surgery requires extracorporeal circulation, which is traumatic and highly risk. Patients who have undergone conventional surgery have many complications and recover slowly (2). Children undergoing vascular intervention are exposed to radiation and contrast agents. The use of radiation must be limited because it may cause irreversible damage (3–5). The emergence of transoesophageal echocardiography (TEE) and its application in interventional treatments can prevent damage from radiation and contrast agents and allow monitoring of the operation and valve opening and closing activities in real time. This study explores the safety and efficacy of percutaneous interventional treatment for children with ASD and PS under the guidance of TEE alone.

## Materials and Methods

### Subjects

Pediatric patients who underwent simultaneous percutaneous interventional treatment for ASD and PS under the guidance of TEE alone at the Dalian Municipal Women and Children's Medical Center (Group) were continuously recruited between March 2015 and March 2019. The inclusion criteria were as follows: (1) patients ≥2 years old who had type II ASDs of the foramen ovale with a diameter >5 mm. The edge of the defect was ≥5 mm from the superior and inferior vena cava, coronary sinus, and pulmonary veins and ≥7 mm from the atrioventricular valve. The diameter of the atrial septum was larger than the diameter of the left atrium side of the selected septal occluder. (2) Patients with concurrent simple PS with a pressure gradient across the pulmonary valve ≥40 mm. The exclusion criteria were as follows: (1) patients with ostium primum ASD or sinus venosus ASD, endocarditis, or hemorrhagic disorder; (2) patients with infundibular pulmonary stenosis, PS accompanied by congenital subvalvular pulmonary stenosis, PS accompanied by supravalvular pulmonary stenosis, severe dysplastic PS, or concurrent diseases that required surgical treatment; and (3) diseases that were contraindications for TEE, such as esophageal stenosis and tonsillitis. A total of 11 children were recruited, including 4 males and 7 females, with an age of 11.9 ± 1.8 years (9.0–15 years) and a body weight of 32.9 ± 8.0 kg (21.0–50.0 kg). Preoperative transthoracic echocardiography (TTE) showed that all patients had type II ASDs of the foramen ovale with a diameter of 12.9 ± 2.7 mm. The patients also had PS with a transvalvular pressure gradient of 54.7 ± 5.8 mmHg (Table 1). Prior to the surgery, all of the patients underwent routine examinations, electrocardiography, anteroposterior and lateral chest X-rays, and TTE to confirm the diagnosis and surgical indications. This study was approved by the medical ethics committee of the Dalian Municipal Women and Children's Medical Center (group). Prior to the operation, the family members of the children all gave informed consent and signed an informed consent form for the operation.

**Table 1:** Clinical data of the patients in this study.

**Item**	
No. of patients	11
Sex (F/M)	7/4
Age (years)	11.9 ± 1.8
Body weight (kg)	32.9 ± 8.0
ASD size (mm)	12.9 ± 2.7
Device size (mm)	16.1 ± 3.1
Balloon size (mm)	24.9 ± 2.8
Preoperative PS gradient (mmHg)	54.7 ± 5.8
Postoperative PS gradient (mmHg)	21.3 ± 1.8
Procedure time (min)	53.0 ± 7.7
Hospital stay (days)	5.1 ± 0.7
Follow-up (years)	3.0 ± 1.4
Success rate	100%

*ASD, Atrial septal defect; PS, pulmonary valvular stenosis*.

### Materials

An Amplatzer septal occluder (AGA Medical Corporation, USA) was used to occlude the ASD. A Philips IE33 device (Philips Medical Systems, Andover, MA, USA) was used for TEE. A BALT balloon (BALT extrusion, France) was used for balloon pulmonary valvuloplasty.

### Surgical Methods

**Figure 1:**
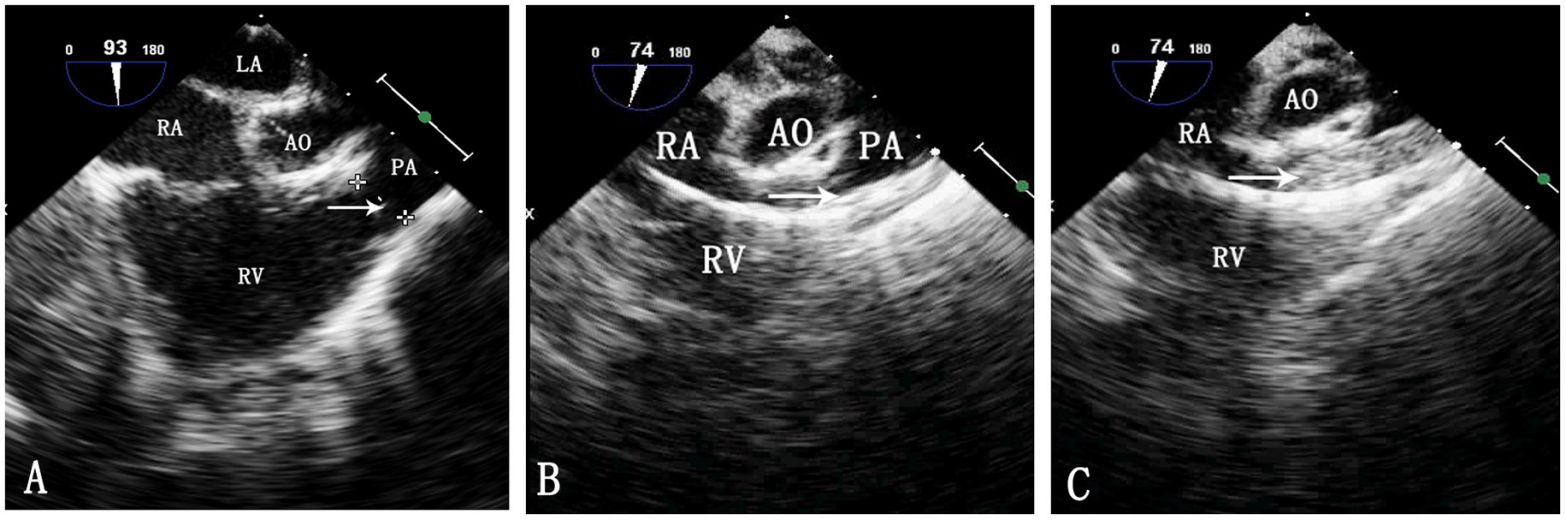
TEE image during pulmonary balloon valvuloplasty. LA, left atrium; RA, right atrium; Ao, aorta; RV, right ventricle; PA, pulmonary artery. **(A)** Measurement of the pulmonary valve annulus by TEE. The arrow shows the pulmonary valve annulus. **(B)** Passing of the catheter through the pulmonary artery. The arrow indicates the catheter. **(C)** Inflation of the balloon. The arrow indicates the balloon.

**Figure 2:**
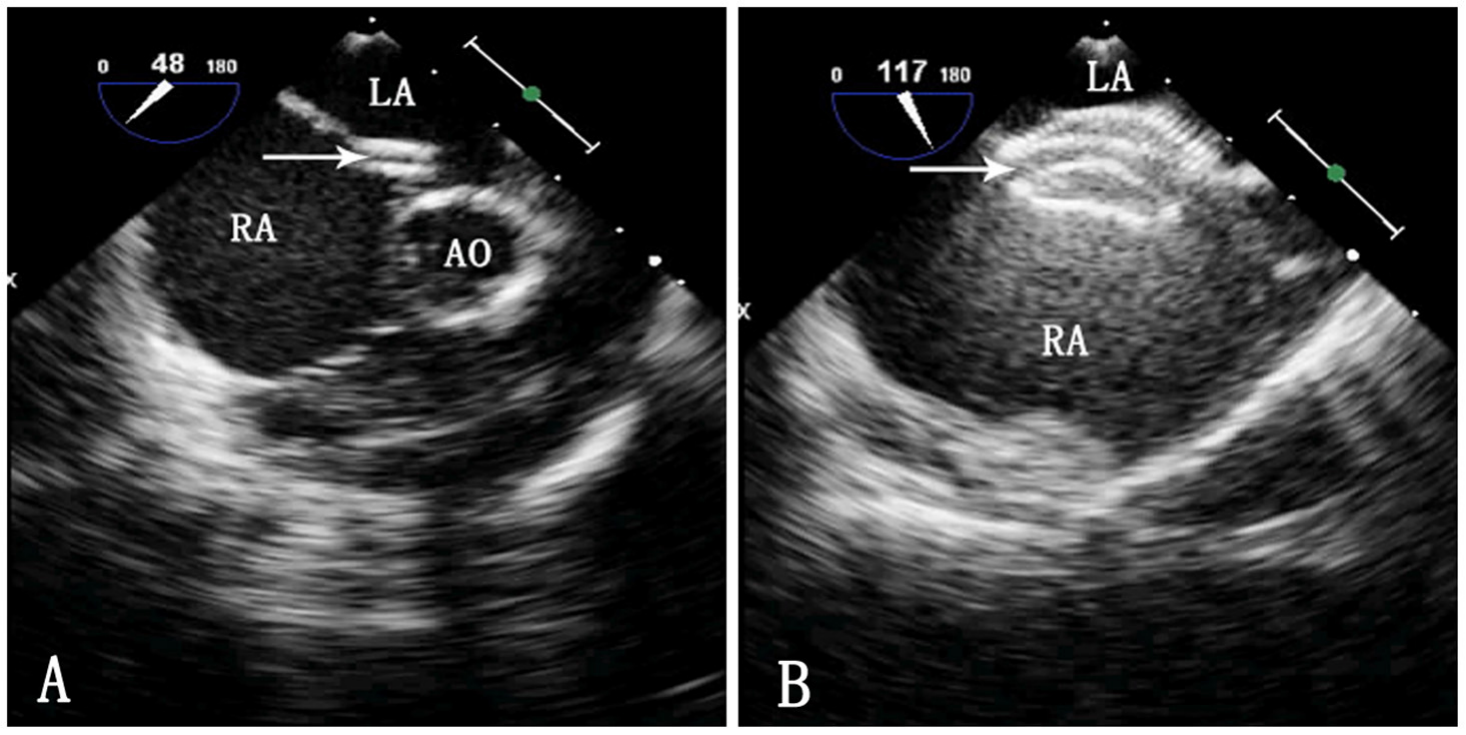
TEE image during ASD occlusion. LA, left atrium; RA, right atrium; Ao, aorta. **(A)** Passing of the sheath through the ASD. The arrow indicates the delivery sheath. **(B)** Morphology of the occluder after release. The arrow indicates the occluder.

The patients fasted for 8 h prior to the surgery, and antibiotics were administered 30 min before the surgery to prevent infection. The trachea was intubated under general anesthesia, and the pediatric TEE probe was placed. The surgical indications were confirmed. The patient was placed in the supine position. The right femoral vein was punctured, and a suitable vascular sheath was placed. A 6F multifunctional catheter and a guide wire were inserted through the vascular sheath and delivered to the right ventricle through the tricuspid valve under TEE guidance. The direction of the catheter and guide wire was adjusted so that they entered the main pulmonary artery through the pulmonary valve. The guide wire entered the left pulmonary artery through the pulmonary valve. After the right ventricle and pulmonary artery pressure was measured through the catheter, the guide wire was replaced with a superhard guide wire, and the pulmonary artery dilatation balloon was delivered along the guide wire. The diameter of the balloon was 1.2–1.4 times the diameter of the pulmonary valve annulus. When ultrasound showed that the head of the balloon had reached the pulmonary valve annulus, the balloon was partially inflated so that the middle of the balloon was located at the pulmonary valve annulus. The balloon and guide wire were fixed. The balloon was inflated to 8–10 atmospheres for a duration of 5–10 s and then quickly deflated. After the balloon was withdrawn, the pressure gradient across the pulmonary valve was measured. If the pressure gradient was still greater than 40 mmHg, a larger balloon was used for another dilatation until the transvalvular systolic pressure gradient was lower than 40 mmHg (Figure 1). The guide wire was retracted to the right ventricle and inserted into the left atrium through the tricuspid valve and the ASD. The ASD delivery sheath was inserted into the left atrium through the guide wire, and the ASD occluder was delivered through the sheath. Under TEE monitoring, the occluder disk at the left atrium was released. The delivery sheath was retracted, and the occluder disk at the right atrium was released. The position and morphology were confirmed to be normal with no deformation or displacement during the push-pull test. When no arrhythmia or shunting at the atrial septum was detected and each valve could open and close normally, the occluder was released, and the delivery system was removed. The bleeding was stopped, and the wound was bandaged with compression (Figure 2).

**Figure 3:**
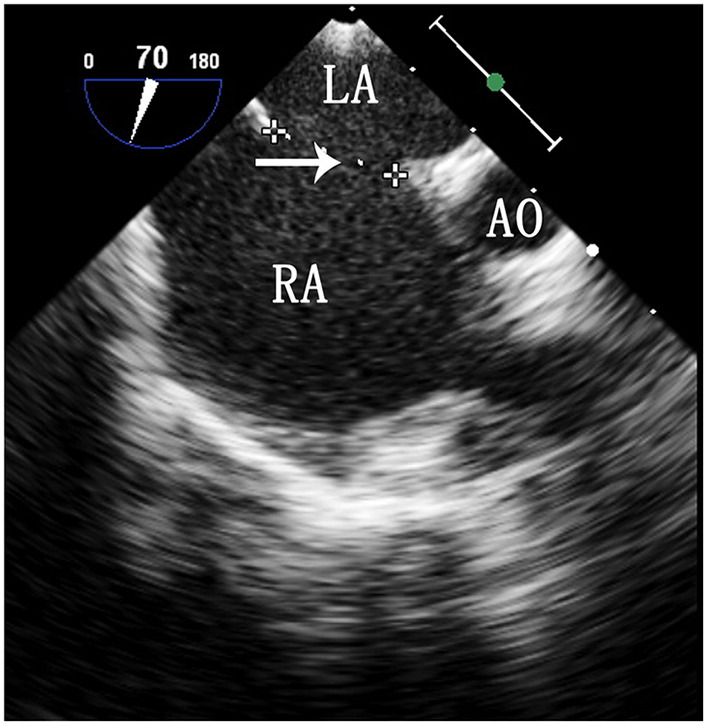
TEE image of the ASD on a two atrial chamber section. LA, left atrium; RA, right atrium; Ao, aorta. The arrow indicates the maximum diameter of the ASD.

**Figure 4:**
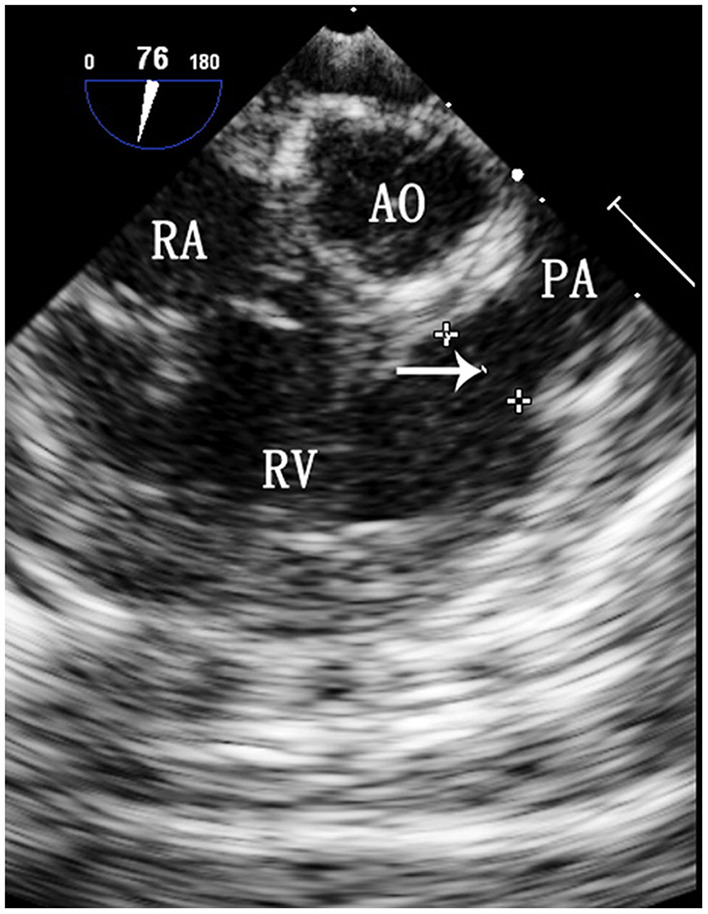
TEE image of the maximum diameter of the pulmonary valve annulus. RA, right atrium; Ao, aorta; RV, right ventricle; PA, pulmonary artery. The arrow indicates the maximum diameter of the pulmonary valve annulus.

Selection of the ASD occluder and PS balloon. ASD occluder: The maximum diameter of the ASD was measured on a four chamber heart section and a two atrial chamber section *via* intraoperative esophageal ultrasound. Generally, we added 2–4 mm to the maximum diameter measured on the two atrial chamber section (Figure 3) to determine the ASD occluder size. Balloon: Esophageal ultrasound was used to simultaneously show the right ventricular outflow tract and pulmonary artery to measure the maximum diameter of the pulmonary valve annulus (Figure 4), and a balloon size 1.2–1.4 times the measured value was used.

### Follow-Up Methods

All of the children were given oral aspirin (3–5 mg/kg/day) for 6 months after surgery and underwent TTE, urinalysis, and electrocardiography at the outpatient clinic at 1, 3, 6, 9, and 12 months after surgery. Thereafter, the patients were followed up every 6 months.

### Statistical Analysis

The data were analyzed using SPSS 20.0 software. Quantitative data are expressed as $\overset{\bar{}}{x}$ ± s. The transvalvular pressure gradients of the pulmonary valve before and after the operation were compared using a paired *t*-test. Differences with *P* < 0.01 were considered statistically significant.

## Results

All 11 patients underwent simultaneous percutaneous interventional pulmonary balloon valvuloplasty and ASD occlusion without radiation or contrast agents under the guidance of TEE alone. One patient had ventricular fibrillation during the operation but was successfully treated with electric cardioversion, and the surgery was completed. The success rate of the surgery was 100%. The operation duration was 53.0 ± 7.7 min (45.0–69 min), and the duration of hospital stay was 5.1 ± 0.7 days (4.0–6.0 days). The transvalvular pressure gradient across the pulmonary valve was 54.7 ± 5.8 mmHg before the operation and 21.2 ± 1.8 mmHg immediately after the operation. The diameter of the ASD occluder was 16.1 ± 3.1 mm (11.0–22.0 mm), and the balloon was 24.9 ± 2.8 mm (20.0–28.0 mm). No shunting at the atrial septum level was detected by ultrasound immediately after the operation. The patients were followed up for 3.0 ± 1.4 years. One patient had mild pulmonary valve regurgitation that did not become aggravated. The position of the ASD occluder was normal. No residual shunting, arrhythmia, or hemolysis was detected. The pressure gradient across the pulmonary valve at 1 month after the operation was 18.5 ± 3.3 mmHg, which was significantly lower than that before the procedure (*P* < 0.01; Table 1).

## Discussion

Congenital heart disease is a common birth defect, and complex congenital heart disease accounts for 2.5–3% of live-born children with congenital heart disease (6). King et al. (7) introduced conventional vascular intervention for closing ASDs in 1976, and this method was successfully applied in clinical practice. In 1982, Kan et al. (8) introduced the conventional vascular interventional balloon dilatation method for the treatment of PS. This method developed rapidly due to its many advantages, including not requiring extracorporeal circulation or a surgical incision (3, 9–11). In 1997, Yip et al. (12) reported the simultaneous treatment of ASD and PS using the conventional vascular intervention method. However, during conventional interventional treatment, children and physicians are exposed to radiation, and contrast agents are required, which increases the damage caused by the treatment (13).

In recent years, echocardiography, mainly TEE and TTE, has developed rapidly. Echocardiography has been widely used in the interventional treatment of congenital heart diseases. Echocardiography does not require radiation or contrast agents and can be used to monitor and guide the operation process in real time. Ewert et al. (14) and Schubert et al. (15) independently reported percutaneous interventional ASD occlusion under the guidance of TEE alone. Moreover, Pan et al. (16) reported their experience with percutaneous interventional ASD occlusion under the guidance of TTE alone. Furthermore, Galal et al. (17) reported percutaneous interventional balloon dilatation of PS under the guidance of TTE alone. However, compared with TTE, TEE can provide clearer images because the TEE probe is directly located on the posterior wall of the left atrium (18). In addition, the TTE image is easily affected by the thickness of the thoracic wall and the air in the lung, making the image less clear, especially in older, obese children. This study aimed to investigate the simultaneous percutaneous interventional treatment of ASD and PS in children under the guidance of TEE alone. This method does not require contrast agents or X-ray. No severe postoperative complications were detected, suggesting that this technique has excellent safety and efficacy for the treatment of this type of complex malformation.

In the interventional treatment of ASD with PS, there is controversy regarding the treatment sequence. Nakasato et al. (19) indicated that PS balloon dilatation should be performed before ASD occlusion. In contrast, Hu et al. (20) suggested that ASD occlusion should be performed before PS balloon dilatation. In our study, all of the patients underwent PS balloon dilatation before ASD occlusion. The reasons for this sequence are that on the one hand, due to the complete obstruction of the right ventricular outflow tract during PS balloon dilatation, the residual ASD can be used as a pressure-reducing valve to relieve pressure and prevent excessive damage to the myocardium; on the other hand, this sequence can reduce interference with the ASD occluder and prevent shifting or falling of the occluder.

Percutaneous interventional treatment for such complex deformities under TEE guidance requires close cooperation between the sonographer and the surgeon, in addition to the high technical requirements for TEE. During treatment using this technology, it is difficult to determine the position of the catheter and balloon because ultrasound can only visualize the tissue on a section-by-section basis, while radiation projects through the tissue.

From our experience with this group of patients, we can make the following recommendations. (1) A medical team with excellent internal and external training should be organized. It is best for the sonographer to have an understanding of the three-dimensional configuration of the heart anatomy, have abundant experience with TEE guidance, and be able to cooperate tacitly with the surgeon. The surgeon should be able to skillfully complete percutaneous ASD occlusion and pulmonary balloon valvuloplasty under the guidance of conventional radiation. The surgery should be completed in a hybrid operating room that allows team members to perform open-heart surgery in an emergency situation to ensure the safety of the operation to the greatest extent. (2) Establishment of the track. After the catheter enters the left atrium through the ASD and the main pulmonary artery through the pulmonary valve, it is difficult to determine the position of the end of the catheter. In such cases, an appropriate amount of saline can be injected through the catheter, and the position of the end of the catheter can be determined using ultrasound contrast agents. In addition, after the catheter enters the right ventricle, the operation should be performed gently to prevent arrhythmia caused by excessive stimulation of the myocardium. One patient had ventricular fibrillation during the establishment of the track, which was successfully treated with electric cardioversion. We believe that ventricular fibrillation was caused by excessive stimulation of the right ventricle by the catheter as the track was established. (3) Balloon dilatation. The balloon should be partially inflated when it passes through the pulmonary valve orifice, with the pressure not exceeding 1 atmosphere. Ultrasound can clearly show whether the balloon is located at the valve annulus. The balloon should be placed in the center of the annulus for dilatation. (4) Release of the occluder. It is important to avoid damage to the mitral valve when releasing the left disk. After the left disk is released, the delivery wire is retracted slowly, and the left disk is closely attached to the atrial septum. The delivery wire is fixed, the delivery sheath is retracted, and the right disk is released. When releasing the occluder, it should encircle the lateral wall of the aorta as much as possible to increase stability.

This study has some limitations. First, the number of patients in this study was small, and the follow-up duration was short. Second, the cost of this treatment is high. In the future, the cost can be reduced by establishing funds and other methods, and the number of patients and the duration of follow-up can be increased.

In addition, in this study, we applied two-dimensional transoesophageal echocardiography (2D TEE) guidance. However, compared with three-dimensional transoesophageal echocardiography (3D TEE), 2D TEE can only show the shape of the ASD on a section-by-section basis and cannot show the complete stereological structure of the ASD. In contrast, 3D TEE can completely display the three-dimensional structure of the ASD from all angles and clearly show the relationship with the surrounding structures; therefore, it can be used to more accurately measure the size of the ASD and show the complete shape after the occluder is released.

Simultaneous percutaneous interventional treatment for children with ASD and PS under the guidance of TEE alone, with PS balloon dilatation followed by ASD occlusion, does not require extracorporeal circulation, incision, or radiation and contrast agents. This method has broad prospects for development and application. However, the medical team performing this procedure must undergo rigorous training, particularly in terms of cooperation between the sonographer and the surgeon, and must clearly understand the indications for surgery.

## Data Availability Statement

The datasets presented in this study can be found in online repositories. The names of the repository/repositories and accession number(s) can be found in the article/Supplementary Material.

## Ethics Statement

The studies involving human participants were reviewed and approved by the Medical Ethics Committee of the Dalian Municipal Women and Children's Medical Center (group). Written informed consent to participate in this study was provided by the participants' legal guardian/next of kin.

## Author Contributions

XL and PW designed the study, performed the experiments, and major contributors to writing the manuscript. XL, PW, YL, QZ, and NW performed the experiments, analyzed the data, and wrote the manuscript. All authors contributed to the article and approved the submitted version.

## Conflict of Interest

The authors declare that the research was conducted in the absence of any commercial or financial relationships that could be construed as a potential conflict of interest.

## Publisher's Note

All claims expressed in this article are solely those of the authors and do not necessarily represent those of their affiliated organizations, or those of the publisher, the editors and the reviewers. Any product that may be evaluated in this article, or claim that may be made by its manufacturer, is not guaranteed or endorsed by the publisher.
